# Laser Powder Cladding of Ti-6Al-4V α/β Alloy

**DOI:** 10.3390/ma10101178

**Published:** 2017-10-15

**Authors:** Samar Reda Al-Sayed Ali, Abdel Hamid Ahmed Hussein, Adel Abdel Menam Saleh Nofal, Salah Elden Ibrahim Hasseb Elnaby, Haytham Abdelrafea Elgazzar, Hassan Abdel Sabour

**Affiliations:** 1 National Institute of Laser Enhanced Sciences (NILES), Cairo University, Giza 12611, Egypt; selnaby@niles.edu.eg; 2 Faculty of Engineering, Cairo University, Giza 12611, Egypt; aahussein41@yahoo.com; 3 Central Metallurgical Research and Development Institute (CMRDI), Helwan 11731, Egypt; adelnofal@hotmail.com (A.A.M.S.N.); h_elgazzar@yahoo.com (H.A.E.); helkordy3000@yahoo.com (H.A.S.)

**Keywords:** laser surface treatment, co-axial laser cladding process, titanium alloys, microhardness, wear resistance

## Abstract

Laser cladding process was performed on a commercial Ti-6Al-4V (α + β) titanium alloy by means of tungsten carbide-nickel based alloy powder blend. Nd:YAG laser with a 2.2-KW continuous wave was used with coaxial jet nozzle coupled with a standard powder feeding system. Four-track deposition of a blended powder consisting of 60 wt % tungsten carbide (WC) and 40 wt % NiCrBSi was successfully made on the alloy. The high content of the hard WC particles is intended to enhance the abrasion resistance of the titanium alloy. The goal was to create a uniform distribution of hard WC particles that is crack-free and nonporous to enhance the wear resistance of such alloy. This was achieved by changing the laser cladding parameters to reach the optimum conditions for favorable mechanical properties. The laser cladding samples were subjected to thorough microstructure examinations, microhardness and abrasion tests. Phase identification was obtained by X-ray diffraction (XRD). The obtained results revealed that the best clad layers were achieved at a specific heat input value of 59.5 J·mm^−2^. An increase by more than three folds in the microhardness values of the clad layers was achieved and the wear resistance was improved by values reaching 400 times.

## 1. Introduction

Titanium alloys are widely used in aerospace, marine and chemical industries owing to their intrinsic properties such as high specific strength, excellent corrosion, oxidation resistance, high-temperature performance and creep resistance [1,2]. However, because of their low hardness and poor tribological properties, the application of titanium alloys is severely constrained under severe wear and friction conditions [3,4,5]. Laser cladding is a promising technique that is widely used to enhance the surface properties of many kinds of metals. The laser cladding process a laser material processing technique in which the laser beam is used to add an alloying material onto a substrate to enhance its performance in service [6,7]. It has also been defined as a surface coating technique, which makes use of cheaper materials with superior physical, mechanical and chemical properties to prolong the component service life, even at elevated temperatures [8]. Conventionally, surface coating processes including High Velocity Oxy-fuel (HVOF), Submerged Arc (SA), Tungsten Inert Gas (TIG), Metal Inert Gas (MIG) and Shielded Metal Arc (SMA) welding suffer from numerous restrictions. Thus, the laser cladding technique offers precise control of the coating on the substrate due to excellent control of the focused laser beam, microstructure control, minimal distortion of the substrate due to localized heating with low energy input, minimal dilution of the clad with the substrate material and time and quality delivery [6,9,10].

In general, the cladding material develops from single ceramic or alloy to multiple ceramics or alloys, although single metals or alloys are rarely used in laser cladding nowadays. Apart from pure metal powders, alloys such as Ni-based, Fe-based, and Co-based alloys are prevalent in laser cladding, especially Ni-based alloys, which exhibit better high-temperature, wear and corrosion resistance properties, and easily bond to the substrate [11,12,13]. One of the most commonly used coating techniques in laser cladding on titanium alloys is the metal matrix composites (MMCs) material (ceramics and metals or alloys) [14,15]. Metals or alloys in the composite material matrix have a function of transition. That is, they act as the binding phases between the ceramic reinforced phases and the matrix, and hence reducing the residual stresses. Owing to the particular properties such as high hardness and excellent resistance to abrasion, tungsten carbide (WC) is extensively used as the reinforcements for iron, aluminum and titanium matrix coatings [16,17,18].

Recent studies regarding the formation of a composite coating on the titanium alloy have principally localized on enhancing the hardness and abrasion wear resistance [19]. Ni-based alloys were frequently used as a binding material for cladding of titanium alloys [20]. In the study of Sun et al. [21,22], laser cladding of Ti-6Al-4V alloy with TiC + NiCrBSi powders was carried out. The results showed that the weight loss of the laser clad layers after the wear test was 11.4% of that of Ti-6Al-4V alloys in air, and 47.9% in vacuum. In the research of Kathuria [23] laser cladding was performed with two different percentages of chromium carbide cermets, i.e., Cr_3_C_2_ (70 wt %.) + NiCr (30 wt %.) and Cr_3_C_2_ (50 wt %.) + NiCr (50 wt %.). The conclusion indicated that the laser cladding with 70 wt % of carbide content displays more cracks and higher hardness compared to that 50 wt % of carbide content. Pure nickel with ceramic phase of B_4_C [11] and TiB_2_ ceramic phase mixed with pure Ti [24] was investigated as well. WC as a reinforcement ceramic was used with different binding metals based alloy such as Mo [25], Co [26] and Ti [18] and it resulted in enhancing the hardness and abrasion resistance of the substrate. WC reinforced Ni-based composite coatings were produced on low carbon steel by laser cladding [27] and on AISI 304 stainless steel [16].

From the literature review, it has been found that the composite of WC + NiCrBSi coating by laser surface engineering improves surface properties such as hardness and wear resistance of numerous engineering materials. However, the published work related to this cladding composite with high content of the WC ceramic on titanium alloys is scarce. Therefore, this work attempts to study the outcome of a hard coating reinforced by 60% WC and 40% NiCrBSi on the Ti-6Al-4V substrate by laser cladding. This high percentage of WC has not yet been studied reflecting the limitation of crack formation because of the tough titanium substrate. Furthermore, it is preferable to increase the wear resistance levels of Ti-6Al-4V in order to extend its service life in applications such as aircraft turbine, engine components, aircraft structural components, etc. Ni-based alloys exhibit better high-temperature and wear and corrosion resistance properties. Because they are easier to bond to the substrate, they were selected with the hard ceramic WC. The challenge is to create hard coating of crack-free, nonporous structure with a high level of hardness and abrasion wear resistance than that already achieved by other studies.

## 2. Materials and Methods

### 2.1. Materials

**Figure 1:**
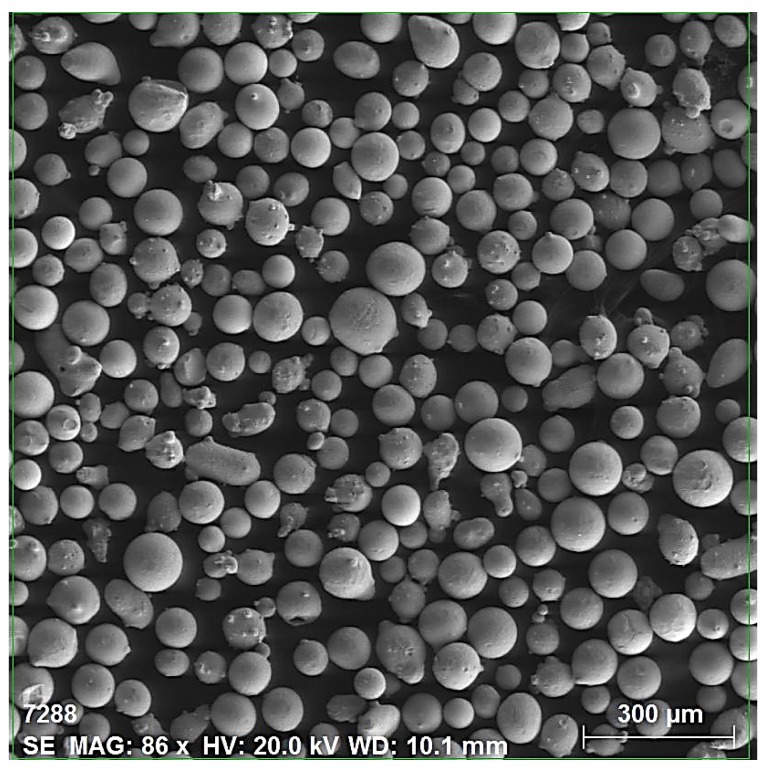
SEM micrographs of the 40NiCrBSi + 60WC blended powder.

A commercial titanium Ti-6Al-4V alloy grade 5 alloy in plate form was received in the annealed condition with dimensions of 300 mm × 300 mm × 5 mm. Small surfaces, used as the substrate were cut measuring 30 mm × 30 mm × 5 mm by using water jet machine. The clad surfaces were ground with emery paper to remove the oxide scale, and rinsed with acetone before starting the laser cladding process. A 60/40 blend of spherical, fused tungsten carbide and a nickel chromium silicon boron matrix was used as the clad material. The powder was used in its commercial as-received blend condition [28]. The NiCrBSi powder has the chemical composition (wt %) of 8.0Cr, 1.6B, 3.5Si, 0.3C, and balance Ni, and, for the WC powder, the chemical composition (wt %) is 3.8C and balance W. Particle size analysis revealed that the particle size ranged from 45 to 125 μm, with a mean particle size of 90 μm. A scanning electron microscopy (SEM) micrograph (BSE mode) is shown in Figure 1.

### 2.2. Cladding System Setup and Deposition Parameters

**Figure 2:**
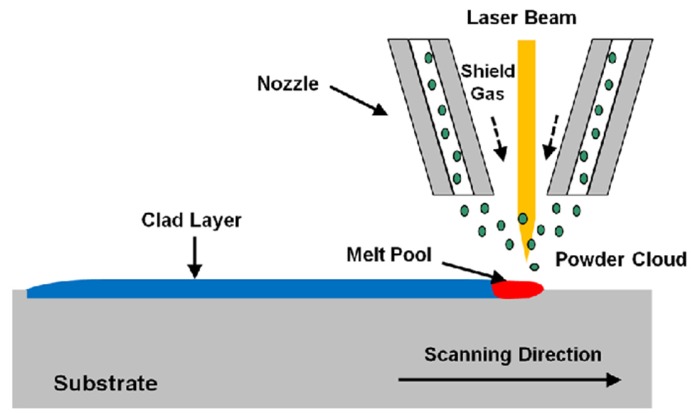
Setup schematic of the coaxial laser cladding process.

A 2.2 KW Nd:YAG CW laser (Rofin-Sinar, Dieburg, Germany) operating at a wavelength of 1064 nm was used for the laser cladding process. It is coupled with a beam delivery system (125 mm collimating lens and a 200 mm focusing lens) and the YC 50 cladding head with coaxial jet nozzle combined with a standard powder feeding system (Precitec KG, Gaggenau, Germany). The delivered laser beam has a Gaussian profile with a spot circular diameter. It is commonplace to operate the laser system out of focus, which provides a wider circular spot to accommodate the materials to be fed into the melt pool generated by the laser beam on the substrate. A single track of laser was performed on the substrate, and then the track width was easily measured. At a defocus distance of 15 mm, a 4 mm circular spot diameter was obtained. The surface cladding process was carried out using argon gas to avert clad oxidation. Figure 2 presents the setup schematic of the coaxial laser cladding process [29]. Clad layers were investigated using optical digital microscope, the transverse cross sections of the clad samples were cut, mounted in conductive bakelite, ground using SiC papers, polished with diamond cloth and finally etched with 92 mL distilled water H_2_O, 6 mL nitric acid HNO_3_, and 2 mL hydrofluoric acid HF (48% concentration) [30] for microscopic analysis. Clads were examined under an Axiotech 30 Optical Microscope (Zeiss, Oberkochen, Germany), in addition to SEM and EDX. Phase constituents of the coating were analyzed using XRD with Cu K α radiation.

#### 2.2.1. Preliminary Attempts of Laser Cladding Process 

Preliminary attempts, shown in Table 1, were carried out to get the suitable laser cladding parameters for good coating quality. Different values of laser power (P), scanning speed and powder flow rate were performed on the clad samples. Preliminary samples were processed at lower laser power of 800 W for the two scanning speeds of 25 and 50 cm·min^−1^ corresponding to a specific heat input of 47.62 and 24 J·mm^−2^, (calculated from the following equation: H_s_ = P/ν·D, where H_s_ is the specific heat input (J·mm^−2^), P is the laser power in watt, ν is the beam scanning speed in mm·s^−1^ and D is the beam diameter in mm [31]), respectively.

The second experiment was conducted based on the observations from the first one, the laser power was increased to 2000 W to obtain higher specific heat input (119 J·mm^−2^) in an attempt to get strong bonding between the clad layer and the substrate. 

#### 2.2.2. Final Attempts of Laser Cladding Process

The final attempts were carried out based on the results obtained from the preliminary ones. Four clad tracks were performed adjacent to each other to achieve the overlapped coating with 50% overlap. A variable laser output power ranging from 600 to 1000 W was used with two corresponding different scanning speeds of 25 and 50 cm·min^−1^, as presented in Table 2. This range of heat input adopted was intended to only melt the NiCrBSi matrix powder without melting the WC particles and to avoid the high percentage of dilution ratio [11,25,27].

### 2.3. Tribological Tests

Microhardness along the depth of the cross-section of the coating was measured by using an HMV micro-hardness tester (SHIMADZU, Kyoto, Japan). The applied load was 980 mN and the loading time was set at 10 s. The indentations were made at 30 µm intervals from the top of clad down into the substrate. Room-temperature dry wear test by means of a TE 79 pin-on-disk type fractional and wear tribometer of the coatings was performed. Samples of 7 mm × 7 mm × 5 mm were used as the pins and a stainless steel alloy of diameter 100 mm and hardness of 65 HRC as the disks. Before each test, the disk was rotated to a defined starting point. The testing conditions were set at; normal loads = 15 N, time = 5 min and fixed sliding at speeds = 800 mm·s^−1^ (150 rpm). The wear test was carried out on the top flat surface of the clad layer. To achieve a flat contact area, the top surface of the chosen clad was carefully ground, using SiC papers and diamond cloth. The wear rate was measured by a weighing method using a sensitive digital balance of accuracy 10^−4^ g.

## 3. Results and Discussion

### 3.1. Microstructural Analysis

#### 3.1.1. Microstructure of the As-Received Specimen

**Figure 3:**
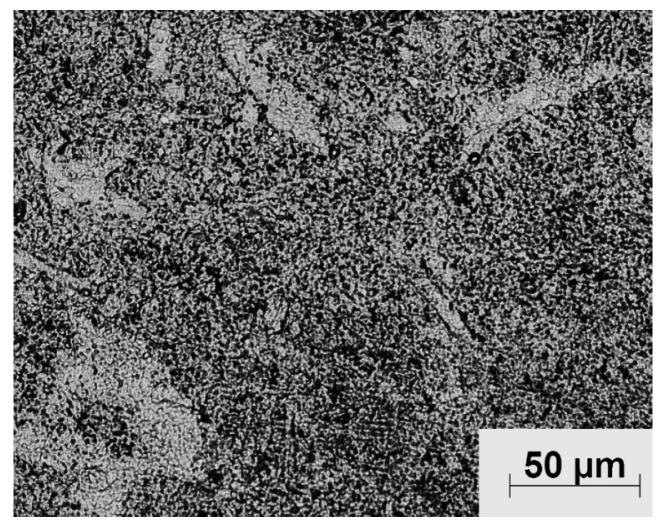
Light micrograph of the as-received Ti-6Al-4V.

Figure 3 shows the cross-sectional microstructure image of the as-received Ti-6Al-4V. The as-received microstructure consists of a fully lamellar microstructure with colonies of alternating laths of α and β phase. Optical microscopy shows the α phase as light regions on the micrograph while the β phase as darker regions.

#### 3.1.2. Microstructure of Preliminary Attempts of the Laser Clad Ti-6Al-4V Specimens 

**Figure 4:**
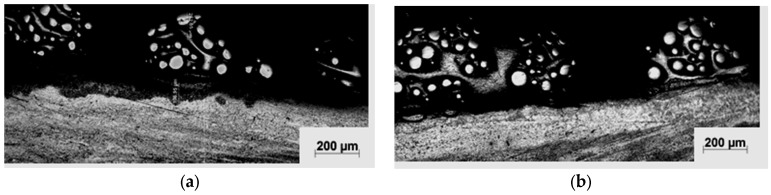

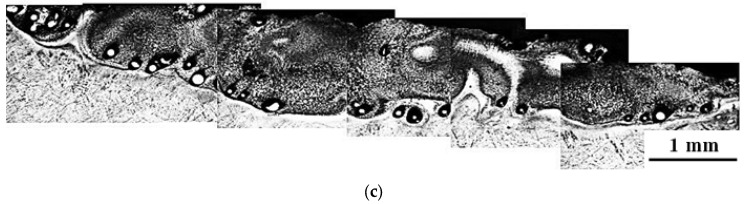
Light micrographs of the cross section of the laser clad samples processed at specific heat input: (**a**) 24 J·mm^−2^; (**b**) 47.62 J·mm^−2^; and (**c**) 119 J·mm^−2^.

Microstructure obtained from preliminary attempts (Figure 4) showed that the powder feed rate of 3 g·min^−1^ might not be suitable for the deposition process, as inconsistency was prevalent. Figure 4a,b shows that the deposited layer is discontinuous along the surface. This is due to the high density of the WC + NiCrBSi powder (6.5–9.5 g·cm^−3^).

In addition, porosities exist between the clad layer and the substrate which imply that clad layers did not provide sufficient bonding to the substrate. In this case, the worn substrate would be exposed on the surface of the fabricated coating and this is not the desired quality that would be acceptable in applications.

**Figure 5:**
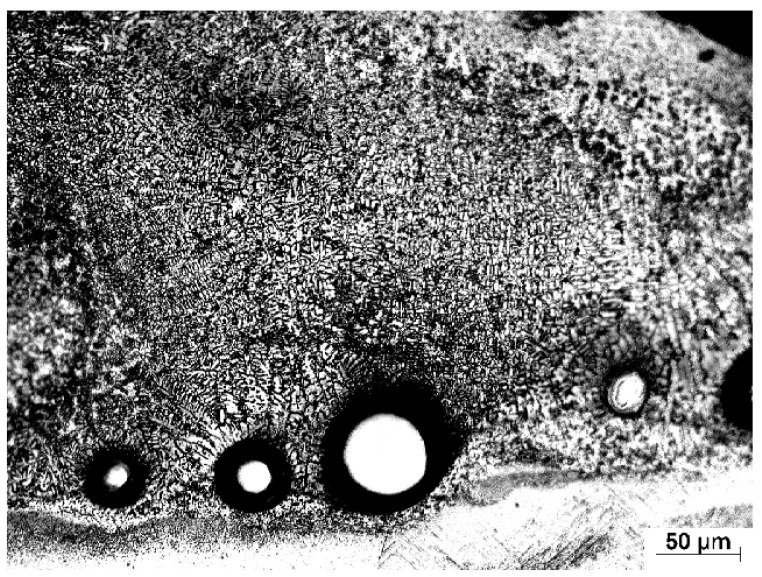
Light micrograph of the cross section of the laser clad samples processed at specific heat input of 119 J·mm^−2^.

When the specific heat input increased to 119 J·mm^−2^, a uniform clad layer was obtained, as shown in Figure 4c, but with intensive melting of the WC particles, which adversely affects the results of hardness and wear resistance. Moreover, it is clear from Figure 5 that WC particles were dissociated, and many needle-like dendrites grew epitaxially from the surface of the partially melted WC particles. This significantly combines the WC particles with the matrix alloy. Some residual WC particles sank to the bottom of the layer. This agrees with the results of other authors [32,33,34]. A high laser energy input might increase the level of dilution of the coating. Thus, the right selection of the laser processing parameters is very important.

#### 3.1.3. Microstructure of Final Attempts of the Laser Clad Ti-6Al-4V Specimens

**Figure 6:**
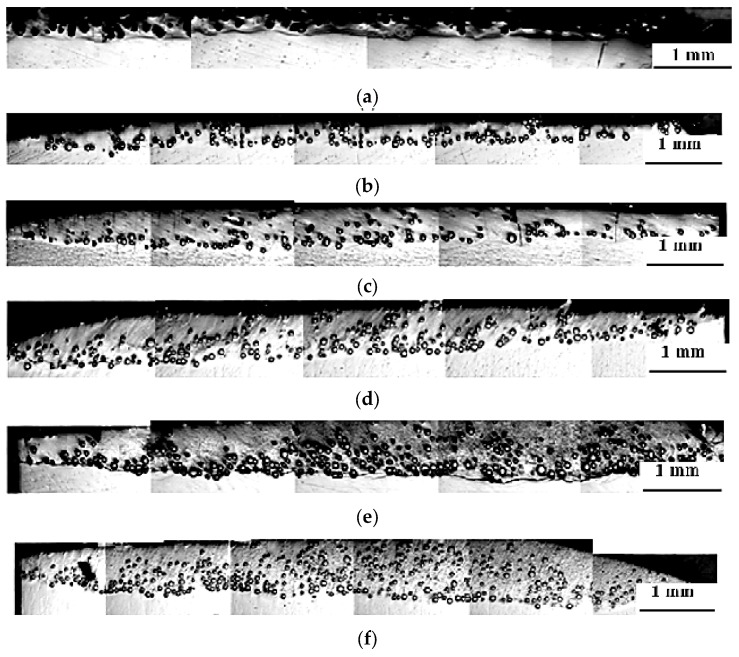
Light micrographs of the cross section (as polished) of the clad layer of Ti-6Al-4V specimens processed at (**a**) 18 J·mm^−2^; (**b**) 24 J·mm^−2^; (**c**) 30 J·mm^−2^; (**d**) 35.71 J·mm^−2^; (**e**) 47.62 J·mm^−2^; and (**f**) 59.5 J·mm^−2^.

It could be perceived in Figure 6 that, at both high laser power and slow scanning speed, the deepest laser clad layer was produced and vice versa. The clad layers had the poorest structure when the supplied specific laser energy was the lowest i.e., 18 J·mm^−2^, large porosities appear in the overlapping regions and the bottom interface of the clad tracks.

A supply of more specific heat input, i.e., 24, 30 and 35.71 J·mm^−2^, results in clad layer without porosities, as shown in Figure 6b–d. When the supplied specific heat input was increased to 59.5 J·mm^−2^, a uniform clad surface was obtained. No cracks at the highest heat input samples were observed. Although the freedom of the high heat input samples from crack formation is encouraged, more work has to be performed in connection with the freedom of clad samples from possibility of crack formation.

**Figure 7:**
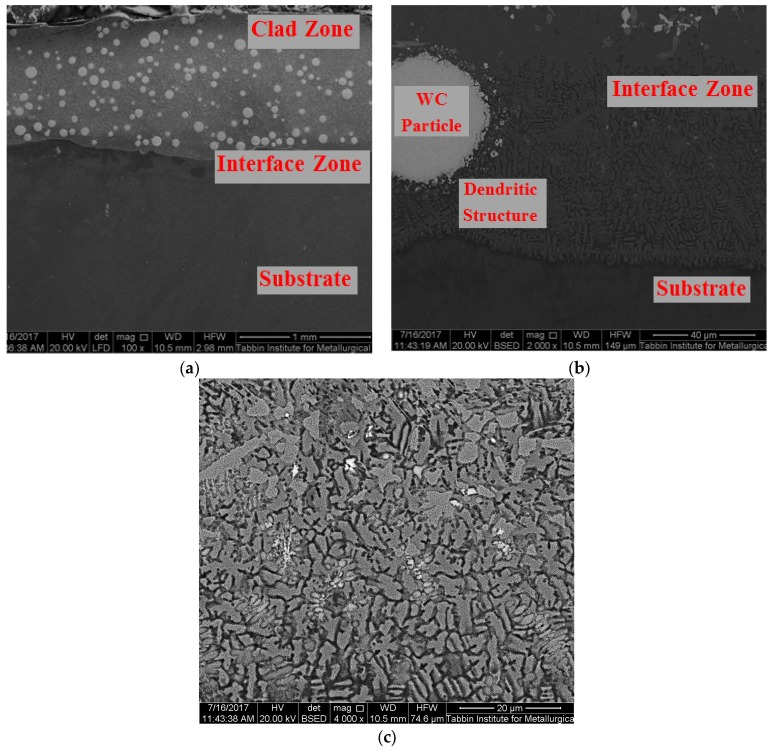
SEM micrographs of sample processed at specific heat input of 59.5 J·mm^−2^: (**a**) whole clad layer; (**b**) interface zone; and (**c**) interface dendritic structure (magnification of 4000×).

Supplementary information of both the clad zone and the interface zone was obtained via SEM. SEM showed that the clad zone consisted of uniform distribution of WC particles with good interfacial bonding between the particle phase and the NiCrBSi matrix, as presented in Figure 7a, whereas the interface zone mainly consisted of epitaxial dendritic structures, occasionally with a few WC particles, as shown in Figure 7b. The dendritic grains were fine, evenly and systemically distributed in the whole coating interface zone (Figure 7c). The same microstructure was observed in both cases, regardless of the specific heat input values.

**Figure 8:**
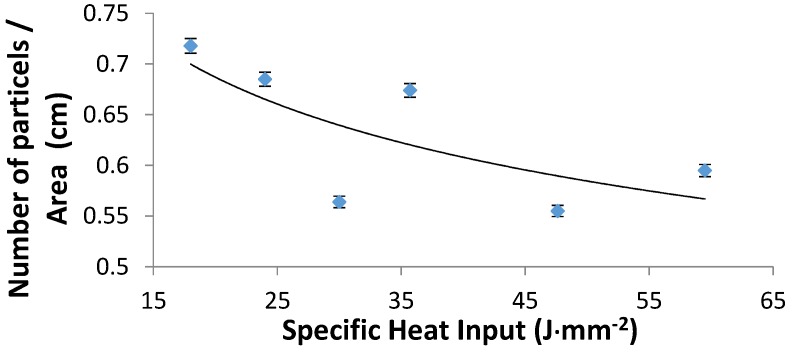
Number of particles per unit area of the clad layer versus the specific heat input.

**Figure 9:**
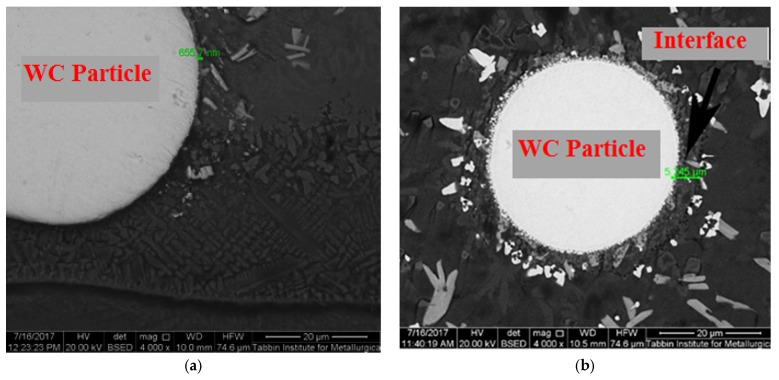
SEM of the interface of the WC particles in case of specific heat input of: (**a**) 18 J·mm^−2^; and (**b**) 59.5 J·mm^−2^.

The number of WC particles per unit clad area (WC particles density) was calculated and plotted vs. the specific heat input, as presented in Figure 8. Each clad layer was divided into five adjacent areas; the number of WC particles was counted in each area; and the total number of particles in the clad surface area was calculated. Figure 8 shows that the general trend is a decrease in the number of particles per unit area with the increase in the specific heat input. The enlargement of the clad area as the specific heat input raises explains the fall of the areal density of the particles. With various laser beam powers and scanning speeds, there were different distributions of WC particles in the layers. The particle distribution in central areas in each sample (see Figure 6) looks more or less uniform. These areas amount to about 75% of the total surface area. From the analysis of cross-sectional characteristics of the WC clad layers, partially dissociated WC hard phase particles were observed in samples processed at specific heat input above 30 J·mm^−2^. It is to be emphasized that higher heat input levels tend to cause partial melting around the interface of WC particle/NiCrBSi matrix (see Figure 9) while the particles themselves are mostly preserved. The thickness of the interface was measured in two different cases; being 0.65 µm in the case of low heat input (18 J·mm^−2^), while, at high heat input (59.5 J·mm^−2^), the interface measured 5.25 µm.

### 3.2. X-ray Diffractograms Compounds Analysis

**Figure 10:**
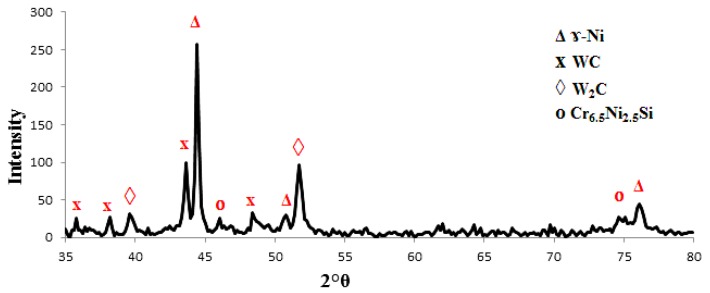
XRD spectra of the 40NiCrBSi + 60WC blended powder.

**Figure 11:**
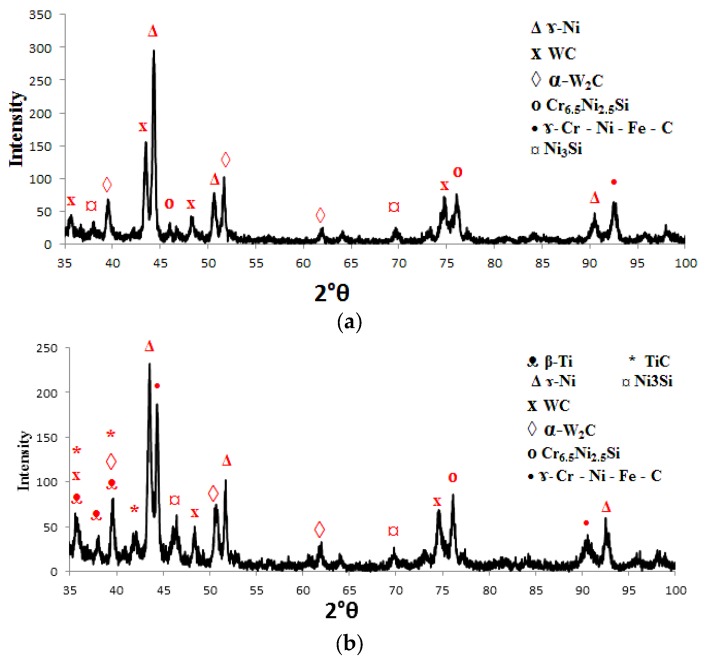
X-ray diffraction spectrum of the clad layer with 40NiCrBSi + 60WC blended powder at specific heat input: (**a**) 18 J·mm^−2^; and (**b**) 59.5 J·mm^−2^ (Ti-6Al-4V as the Substrate).

Figure 10 shows the XRD spectra for the used powder. According to the XRD results of the clad samples shown in Figure 11, the matrix phases in the metal matrix coating (MMC) layers consist mainly of β-Ti, Ni, TiC, WC and W_2_C besides the existence of β-Ti and α-Ti phases in the substrate material. It appears that the effect of the Ni content is to stabilize the β-phase of Ti in the clad layer and α-Ti disappears from the matrix. The existence of β-Ti is preferred compared to α-Ti as it improves the effective bonding strength of the laser fabricated MMC coating [32].

Another observation was that there are other intermetallic compounds or matrix phases formed in the clad layer, such as Ni_3_Si, Cr-Ni-Fe-C and Cr_6.5_ Ni_2.5_Si. The formation of the new clad phase depends on the “wettability” of the contact surface, chemical reactions between the interaction phases, nucleation and growth properties of each phase, and diffusion rates of the reacting species through the interlayer [35,36].

For the range of the specific heat input from 18 to 30 J·mm^−2^, no presence of the TiC phase was observed and this means that no chemical reactions between the interaction phases occurred. With higher specific heat input, i.e. more than 30 J·mm^−2^, there was adequate heat input level to partially dissociate the WC particles. These results are in accordance with Wu et al. [27], Guojian et al. [26] and Farayibi [18].

**Figure 12:**
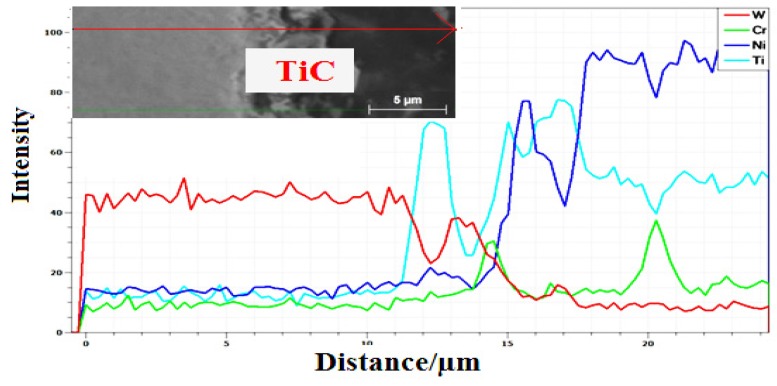
The EDAX compositional line scan of the interface of WC phase for sample processed at 59.5 J·mm^−2^.

Figure 12 shows an energy dispersive analysis by X-ray (EDAX) linescan across the WC particle towards the matrix. The variation of elemental compositions across the line scan complements the phases identified. As the scan passed from the core of the WC particle, the scan observed for W is intense with no trace of other elements. As the scan reaches the TiC layer around the particle edge (identified by XRD), the intensity of Ti becomes the strongest reflection. The intensity of W is negligible in the TiC reaction layer region (Ti + WC → TiC + W). As the scan continues into the composite matrix region, it is fully filled by the NiCrBSi matrix.

### 3.3. Measurements of the Clad Layer Thickness

**Figure 13:**
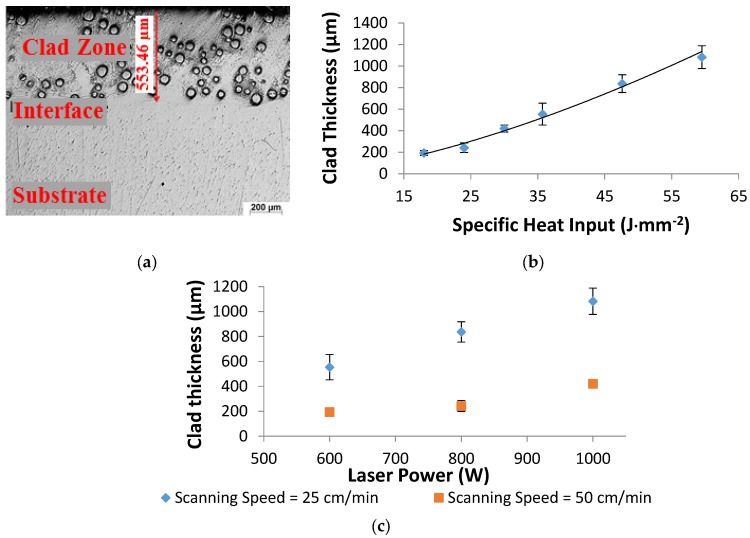
(**a**) Measurement of the clad depth; and the clad depths: (**b**) vs. the specific heat input; and (**c**) vs. laser powers for two laser scanning speeds 25 and 50 cm·min^−1^.

The clad thickness was evaluated by means of optical microscope under a magnification of 50×, from the highest point of the clad layer to the deepest point at the interface in the clad zone, as shown in Figure 13a. The increase in the clad thickness conferring to the high specific heat input is presented in Figure 13b. The thickness of the shallowest clad layer was 195 µm and was associated with the lowest heat input (18 J·mm^−2^), while the deepest clad layer was 1083 µm and was achieved with the highest heat input value (60 J·mm^−2^). The present data also recorded a shallower clad zone accompanying the high laser scanning speeds, as shown in Figure 13c, and the opposite occurred at slower scan speeds. This is understood as high laser scanning speed leads to less energy being applied to the substrate.

### 3.4. Dilution Ratio and Microhardness Profiles

**Figure 14:**
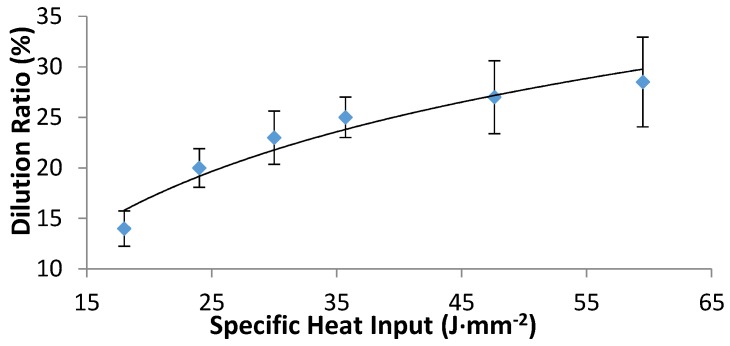
Dilution ratio versus the specific heat input.

The dilution ratio was calculated based on the clad layer geometry [37,38]. It is defined as the ratio of the clad depth (D_c_) in the substrate over the total clad height (T_c_), according to the following equation: D_R_ = D_c_/T_c_, where T_c_ is the summation of the clad height and the clad depth. Figure 14 shows that the dilution ratio of the laser clad layer increases with the increase in the specific heat input, which is in accordance with Pang [25].

#### Microhardness Profiles

**Figure 15:**
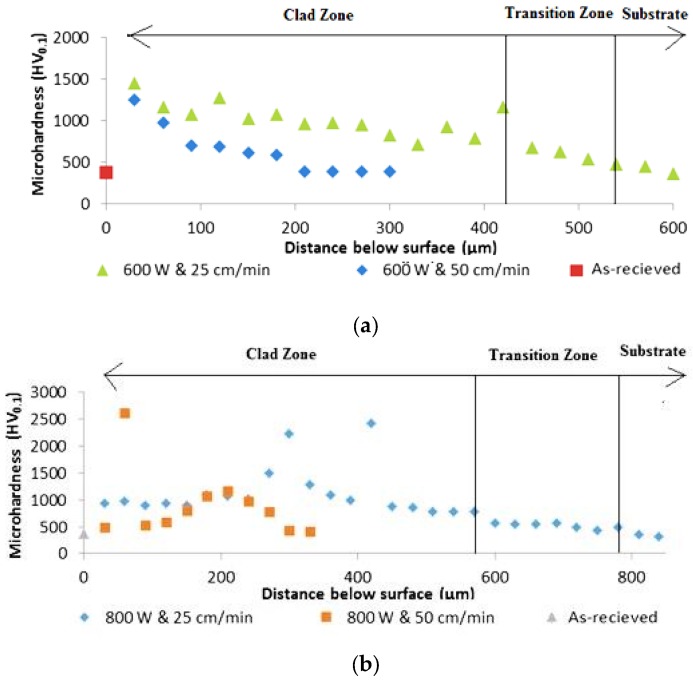

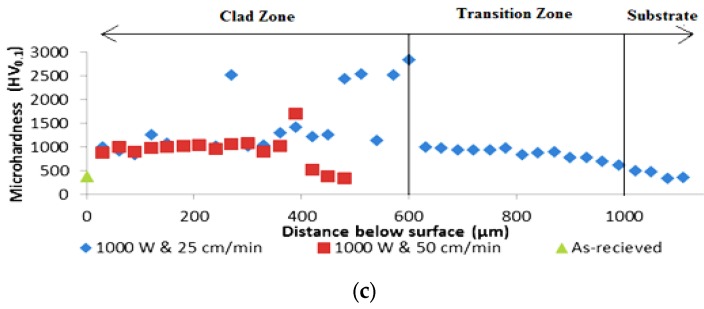
Microhardness profiles of samples processed at laser power of: (**a**) 600 W; (**b**) 800 W; and (**c**) 1000 W, for the two corresponding scanning speeds.

Figure 15 displays the microhardness distribution profiles of samples processed at constant laser power and the two different laser scanning speeds. Clearly, the hardness level can be easily doubled or tripled down to ~400 µm below the surface. 

The microhardness (380 HV) of the lamellar α + β microstructure is the lowest in the as-received samples. However, at the clad layer, the hardness value increased due to the high content of WC in the clad powder with the NiCrBSi matrix. The hardness of the clad composite matrix is enhanced by the uniform distribution of the WC precipitates in the NiCrBSi solid solution. The reduction of the hardness in the interface zone may be attributed to dilution of the clad region content with the Ti substrate.

The microhardness of composite matrix lies between 630 and 1400 HV for samples processed at lower laser scanning speed. However, at higher laser scanning speed, the microhardness lies between 900 and 1600 HV, which is much higher than what had been recorded before [18,25]. The increase in the microhardness at lower laser scanning speed could be related to the more uniformity of the coating as a result of the higher number of WC particles per unit area.

The results show that the clad layer prepared with a laser power of 1000 W exhibits the highest hardness with a mean value of 1195 HV, while the hardness decreases with the decrease in laser power (600 W) with a mean value of 806 HV.

**Figure 16:**
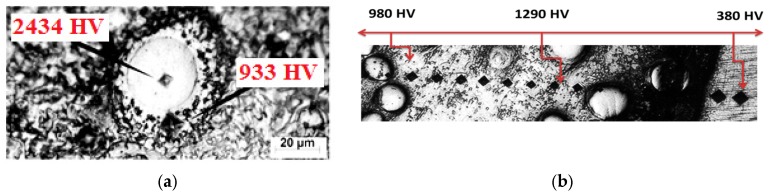
(**a**) High hardness value inside the WC particle; and (**b**) microhardness variations along the clad layer toward the substrate.

There was a non-uniform increase in the hardness values from the clad surface towards substrate due to the distribution of the WC particles inside the NiCrBSi matrix (see Figure 16). Extra high hardness values refer to the indentations through WC particles.

These results seem to be in contradiction with previous studies [25], which demonstrate that an increase in dilution ratio will decrease the microhardness values of the clad layers on WC specimens. The microhardness mean values increase with the increase in the dilution ratio. For example, at the lowest and highest dilution ratios of 18% and 28.8%, the mean values of microhardness increase from 806 to 1195 HV, respectively. This could be attributed to the preservation of the WC particles in the NiCrBSi matrix due to the appropriate specific heat input. This range of specific heat input was sufficient to melt the NiCrBSi matrix and small thickness from the Ti-6Al-4V substrate and obtain a strong bonding between the coating matrix and the substrate.

### 3.5. Wear Resistance

Ti-6Al-4V alloys, owing to their relatively poor wear resistance, have restricted applications in the areas associated with wear. Although such limitations on Ti-6Al-4V cannot be totally eliminated, the effects can be reduced by the use of suitable laser surface MMC coating. The wear properties of the composite clad layer and bare commercial Ti-6Al-4V sample were comparatively investigated.

**Figure 17:**
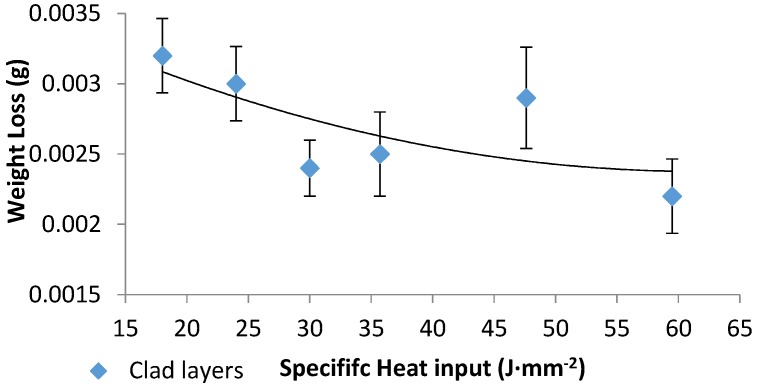
Clad layer weight loss versus the specific heat input.

In this study, an increase in the microhardness values (for example, 1200 vs. 380 HV of the base alloy) was achieved; the hardness of the surface after cladding was increased by more than three folds. The wear resistance of the laser clad layers was clearly increased, consensus with previously studies [18,26,27], as exposed in Figure 17. Even though the weight loss data of the clad samples show some similarity in their values, one cannot ignore their levels compared to the as-received weight loss levels (0.0022 vs. 0.781, respectively). In comparison with the worn surface of the as-received sample, the wear resistance of the MMC coating on Ti-6Al-4V substrate was improved by values reaching 400 times, corresponding to different heat input and WC particles density. The improvement in the wear resistance is double the improvement achieved in previous studies [18,25]. The laser cladding of NiCrBSi-WC metal matrix composite thus offers a remarkable improvement in the wear resistance and performance of Ti-6Al-4V alloy.

## 4. Conclusions

1. Deposition of 60% WC-40% NiCrBSi metal matrix composite layer on Ti-6Al-4V alloy was achieved by coaxial laser surface cladding on the alloy substrate with metallurgical bond. The obtained clad layer on the titanium alloy was divided into three zones according to different microstructure: clad, interface and the substrate zones.
2. In the clad zone, WC particles were uniformly embedded in the NiCrBSi matrix, whereas the interface zone mainly consisted of epitaxial dendritic structures with a few WC particles.
3. The higher heat input levels caused partial melting around the interface of WC particle/NiCrBSi matrix, while the particles themselves were mostly preserved.
4. The thickness of the shallowest clad layer was 195 µm, and was associated with the lowest heat input (18 J·mm^−2^), while the deepest clad layer was 1083 µm and was achieved with the highest heat input value (60 J·mm^−2^).
5. The matrix phases in the metal matrix coating (MMC) layers consist mainly of β-Ti, Ni, TiC, WC and W_2_C besides the existence β-Ti and α-Ti phases in the substrate material. For the range of the specific heat input from 18 to 30 J·mm^−2^, no presence of the TiC phase was observed, while at higher specific heat input, partially dissociation of the WC particles happened due to the adequate heat input level which causes that chemical reaction between the interaction phases.
6. The dilution ratio of the laser clad layers was found to be directly proportional to the incident laser heat input. The microhardness values of the clad NiCrBSi-WC MMC layer on Ti-6Al-4V alloy were found to be directly proportional to the increase in the dilution ratio as well. The microhardness values were increased by three folds over that recorded for the as-received base alloy (1200 vs. 380 HV).
7. After performing the pin-on-disk wear test on the clad layers, the wear resistance was observed to be increased by 400 times over the wear resistance of the as-received alloy.
8. The optimum condition from the parameter window was at the highest specific heat input (59.5 J·mm^−2^), the best clad layer of uniformly distributed WC particles along the clad layer, and nonporous and crack free layer was achieved. It has the highest mean microhardness value (1200 HV) and a sufficient improvement in the wear resistance as well.

In brief, this study has highlighted the substantial and profitable increase in the wear resistance of titanium alloys owing to their poor tribological properties, which limit their applications.

## Figures and Tables

**Table 1:** Preliminary attempts parameters for laser cladding process.

Laser Processing Parameter	First Attempt	Second Attempt
Laser power (W)	800	2000
Scanning speed (cm·min^−1^)	25, 50	25
Powder flow rate (g·min^−1^)	3	3
Feeding gas (L·min^−1^)	2	2
Shielding gas (L·min^−1^)	10	10

**Table 2:** Final attempts parameters for laser cladding process.

Laser Cladding Parameters	Parameter Value
Laser power	600, 800, 1000 W
Beam scanning speed	25, 50 cm·min^−1^
Powder flow rate	6 g·min^−1^
Feeding gas	3 L·min^−1^
Shielding gas	15 L·min^−1^
Defocus distance	15 mm
Beam diameter	4 mm

## References

[1] Li J., Chen C., Squartini T., He Q. (2010). A study on wear resistance and microcrack of the Ti_3_Al/TiAl + TiC ceramic layer deposited by laser cladding on Ti-6Al-4V alloy. Appl. Surf. Sci..

[2] Kartal G., Timur S., Urgen M., Erdemir A. (2010). Electrochemical boriding of titanium for improved mechanical properties. Surf. Coat. Technol..

[3] Savalani M.M., Ng C.C., Li Q.H., Man H.C. (2012). In situ formation of titanium carbide using titanium and carbon-nanotube powders by laser cladding. Appl. Surf. Sci..

[4] Tian Y.S., Chen C.Z., Li S.T., Huo Q.H. (2005). Research progress on laser surface modification of titanium alloys. Appl. Surf. Sci..

[5] Tian Y.S., Chen C.Z., Chen L.X., Huo Q.H. (2006). Microstructures and wear properties of composite coatings produced by laser alloying of Ti-6Al-4V with graphite and silicon mixed powders. Mater. Lett..

[6] Ion J.C. (2005). Laser Processing of Engineering Materials: Principles, Procedure and Industrial Application.

[7] Jang J.H., Joo B.D., Van Tyne C.J., Moon Y.H. (2013). Characterization of deposited layer fabricated by direct laser melting process. Met. Mater. Int..

[8] Zhang D., Cai Q., Liu J., He J., Li R. (2013). Microstructural evolvement and formation of selective laser melting W-Ni-Cu composite powder. Int. J. Adv. Manuf. Technol..

[9] Mok S.H., Bi G., Folkes J., Pashby I. (2008). Deposition of Ti-6Al-4V using a high power diode laser and wire, Part I: Investigation on the process characteristics. Surf. Coat. Technol..

[10] Sachdev A.K., Kulkarni K., Fang Z.Z., Yang R., Girshov V. (2012). Titanium for automotive applications: Challenges and opportunities in materials and processing. JOM.

[11] Song R., Li J., Shao J.Z., Bai L.L., Chen J.L., Qu C.C. (2015). Microstructural evolution and wear behaviors of laser cladding Ti_2_Ni/α(Ti) dual-phase coating reinforced by TiB and TiC. Appl. Surf. Sci..

[12] Chen Y., Wang H.M. (2004). Rapidly solidified MC carbide morphologies of a pulsed laser surface alloyedɤ-TiAl intermetallic with carbon. Scr. Mater..

[13] Du B., Zou Z., Wang X., Qu S. (2008). Laser cladding of in situ TiB_2_/Fe composite coating on steel. Appl. Surf. Sci..

[14] Pu Y., Guo B., Zhou J., Zhang S., Zhou H., Chen J. (2008). Microstructure and tribological properties of in situ synthesized TiC, TiN, and SiC reinforced Ti_3_Al intermetallic matrix composite coatings on pure Ti by laser cladding. Appl. Surf. Sci..

[15] Yang Y., Zhang D., Yan W., Zheng Y. (2010). Microstructure and wear properties of TiCN/Ti coatings on titanium alloy by laser cladding. Opt. Lasers Eng..

[16] Varela J.A., Amado J.M., Tobar M.J., Mateo M.P., Yañez A., Nicolas G. (2015). Characterization of hard coatings produced by laser cladding using laser-induced breakdown spectroscopy technique. Appl. Surf. Sci..

[17] Dubourg L., Ursescu D., Hlawka F., Cornet A. (2005). Laser cladding of MMC coatings on aluminium substrate: Influence of composition and microstructure on mechanical properties. Wear.

[18] Farayibi P.K., Folkes J., Clare A., Oyelola O. (2011). Cladding of pre-blended Ti-6Al-4V and WC powder for wear resistant applications. Surf. Coat. Technol..

[19] Zhang C., Fujii M. (2016). Tribological Behavior of Thermally Sprayed WC Coatings under Water Lubrication. Mater. Sci. Appl..

[20] Haldar B., Saha R., Agarwal P.S., Chattopadhyay A.B. Laser Cladding of In-situ TiB, TiC and TiN Reinforced Ni-Ti MMC Coating on Ti-6Al-4V for Improving Tribological Performance. Proceedings of the 4th International & 25th All India Manufacturing Technology, Design and Research Conference (AIMTDR 2012).

[21] Sun R.L., Lei Y.W., Niu W. (2009). Laser clad TiC reinforced NiCrBSi composite coatings on Ti-6Al-4V alloy using a CW CO_2_ laser. Surf. Coat. Technol..

[22] Liu X.B., Meng X.J., Liu H.Q., Shi G.L., Wu S.H., Sun C.F., Wang M.D., Qi L.H. (2014). Development and characterization of laser clad high temperature self-lubricating wear resistant composite coatings on Ti-6Al-4V alloy. Mater. Des..

[23] Kathuria Y.P. (2001). Nd-YAG laser cladding of Cr_3_C_2_ and TiC cermets. Surf. Coat. Technol..

[24] Lin Y., Lei Y., Fu H., Lin J. (2015). Mechanical properties and toughening mechanism of TiB_2_/NiTi reinforced titanium matrix composite coating by laser cladding. Mater. Des..

[25] Pang W., Man H.C., Yue T.M. (2005). Laser surface coating of Mo-WC metal matrix composite on Ti6Al4V alloy. Mater. Sci. Eng. A.

[26] Xu G., Kutsuna M., Liu Z., Sun L. (2006). Characteristic behaviours of clad layer by a multi-layer laser cladding with powder mixture of Stellite-6 and tungsten carbide. Surf. Coat. Technol..

[27] Wu P., Zhou C.Z., Tang X.N. (2003). Microstructural characterization and wear behavior of laser cladded nickel-based and tungsten carbide composite coatings. Surf. Coat. Technol..

[28] Oerlikon Group—Balzers, Metco, Barmag, Neumag, Graziano, Fairfield << Oerlikon Corporate. https://www.oerlikon.com/en/.

[29] Powell J., Henry P.S., Steen W.M. (1988). Laser Cladding with Preplaced Powder: Analysis of Thermal Cycling and Dilution Effects. Surf. Eng..

[30] Metallographic Etching. http://www.metallographic.com/Technical/Etching.htm.

[31] Steen W.M., Mazumder J. (2010). Laser Material Processing.

[32] Zhang S., Wu W.T., Wang M.C., Man H.C. (2001). In-situ synthesis and wear performance of TiC particle reinforced composite coating on alloy Ti6Al4V. Surf. Coat. Technol..

[33] Choudhury A.R., Ezz T., Chatterjee S., Li L. (2008). Microstructure and tribological behaviour of nano-structured metal matrix composite boride coatings synthesized by combined laser and sol-gel technology. Surf. Coat. Technol..

[34] Wu P., Chen X.L., Jiang E.Y. (2003). Morphology and gradient distribution of WC phase in laser-clad NiCrBSiC-WC composite layers. Phys. Status Solidi Appl. Res..

[35] Man H.C., Zhang S., Cheng F.T., Yue T.M. (2001). Microstructure and formation mechanism of in situ synthesized TiC/Ti surface MMC on Ti-6Al-4V by laser cladding. Scr. Mater..

[36] Wang X.H., Zhang M., Liu X.M., Qu S.Y., Zou Z.D. (2008). Microstructure and wear properties of TiC/FeCrBSi surface composite coating prepared by laser cladding. Surf. Coat. Technol..

[37] Noskov A.I., Gilmutdinov A.K., Yanbaev R.M. (2017). Effect of coaxial laser cladding parameters on bead formation. Int. J. Miner. Metall. Mater..

[38] Schneider M.F. (1998). Laser Cladding with Powder, Effect of Some Machining Parameters on Clad Properties.

